# Making sense of estimates of health aid from China

**DOI:** 10.1136/bmjgh-2019-002261

**Published:** 2020-02-13

**Authors:** Kaci Kennedy McDade, Wenhui Mao

**Affiliations:** Global Health Institute, Duke University, Durham, North Carolina, USA

**Keywords:** health policy

## Introduction

The global aid landscape is rapidly changing. So-called ‘emerging’ or ‘non-traditional’ donors (ie, those that have only recently substantially stepped up their development finance support) are pushing the boundaries of existing aid practices. Among such non-traditional donors, China stands out as a major player because of its recent high-profile commitments, like the Belt and Road Initiative, and its major role in establishing new development finance institutions, such as the Asian Infrastructure Investment Bank. Although China is often thought of as a new donor, this categorisation is somewhat misleading: China has provided aid to fellow low-income and middle-income countries for decades. However, China’s role as a major financier of and leader in global development is a relatively recent phenomenon.

Health aid played a major role in China’s external aid even in its earliest days. Chinese medical teams were among China’s flagship foreign policy efforts; China dispatched its first medical team to Algeria in 1963.^1^ Chinese health aid typically involves transferring experience and lessons learnt from domestic successes. For example, based on China’s own success in controlling malaria, it sent medical teams to malaria endemic countries to advise on malaria control. Its aid primarily focuses on dispatching medical teams, providing in-kind medical equipment and drugs, building health infrastructure and assisting in the prevention and control of infectious diseases, particularly malaria.^2^

In the last few years, health has played a much more prominent role in China’s foreign engagement. To complement the Belt and Road Initiative’s focus on infrastructure, China signed a memorandum of understanding with the World Health Organization for a ‘Health Silk Road’.^3^ Health was also front and centre at the latest Forum on China-Africa Cooperation (FOCAC): global health was one of eight main initiatives committed to by President Xi and African leaders.^4^ On the sidelines of the latest FOCAC, China’s first lady, Peng Liyuan, led a side event with African first ladies on combatting HIV/AIDS in Africa.^5^

## Why we need estimates of aid from China

Given China’s expanding role in the development finance space, there have been many attempts by researchers to better understand the volume and focus of Chinese aid. Such estimates are needed because unlike the 30 traditional donors, who must publish detailed aid information as part of their membership in the Organization for Economic Cooperation and Development (OECD) Development Assistance Committee (see http://www.oecd.org/dac/development-assistance-committee/), China makes very little information available to the public. To give one example, China ranked last in the 2018 Publish What You Fund Aid Transparency Index, an ‘independent measure of aid transparency among the world’s major development agencies’.^6^

There are two documents that the Chinese government released in recent years that give some glimpse into its aid approach: the foreign aid white paper of 2011^1^ and the foreign aid white paper of 2014.^2^ These white papers give a high-level overview of the aid landscape, the regions that receive the greatest funds, and some examples of key projects and priorities. While a step in the right direction, the white papers lack disaggregated, detailed information, particularly at the sector level. Additionally, the very definition of foreign aid in China differs from the definition used by other donors, and therefore the reported figures in the white papers are not comparable with those of other donors. If we want to understand the full scope of China’s aid portfolio, and how it compares to the portfolios of other donors, we need a better measuring stick.

Current estimates of the total annual volume of health aid typically exclude donors like China, given the unavailability of Chinese data and the difficulties comparing estimates of Chinese health aid with data on health aid from other donors. Given the lack of official data on China’s aid, several academics have developed estimates of the scope of China’s global development footprint.

## Differences between existing estimates of health aid from China

There are several estimates that target health aid specifically (see table 1 for overview of five identified studies, one of which was recently published in *BMJ Global Health*). There are generally two types of estimates: those that take aggregate financial data and try to identify how much of it can be considered aid (‘top-down’), and those that try to identify individual financial flows and total them up to get an overarching estimate (‘bottom-up’). Several of these estimates draw from broader development estimates and datasets, and therefore share many of the same characteristics and limitations as their origin data. However, several unique factors emerge among health aid estimates that must be taken into account when determining how to appropriately characterise or use the estimates:

**Table 1:** Overview of Chinese aid for health estimates

Characteristic	Detail	China’s distinctive engagement in global health^11^	China’s engagement with development assistance for health in Africa^12^	China’s role as a global health donor in Africa: what can we learn from studying under reported resource flows?^13^	China’s silk road and global health^14^	Tracking development assistance for health from China, 2007–2017^15^
Definition and scope of health aid*						
**Flow**	Official development assistance (ODA)- like	N	Y	Y	Y	N
	Other official finance (OOF)-like	N	Y	Y	Y	N
	Vague (cannot be determined as ODA or OOF)	N	Y	Y	Y	N
	Official investment	N	N	N	Y	N
	Other	Refers to ‘health aid’ although no definition provided	N	N	N	Dsbursement of in-kind or financial resources (grants, interest-free/concessional loans) to low-income or middle-income countries
**Flow type**	Disbursements	Unclear	Y†	Y†	Unclear	Y
	Commitments	Unclear	N	Y	Unclear	N
	Pledges	Unclear	Y	N	Unclear	N
**Sector**	Health	Unclear	Y	Y	Y	Unclear
	Population and reproductive health	Unclear	Y	Y	Y	Unclear
	Water, sanitation and hygiene	Unclear	Y	Y	N	Unclear
	Other	Unclear	Key word search conducted to identify health projects in other sectors (eg, government and civil society)	All social sector projects were manually inspected for health-related projects	N	Included flows that were for the ‘primary purposes of improving or maintaining health’
**Channel**	Bilateral	Y	Y	Y	Y	Y
	Multilateral	Unclear	N	N	Y	Y
**Time and geographic scope**						
**Time period**		2007–2011	2000–2013	2000–2012	2008–2013	2007–2017
**Geography**		Africa	Africa	Africa	Global	Global
**Results: level of disaggregation**						
**Project count**		Y	Y	Y	Y	N
**Recipient country**		Y	Y	Y	N	Y
**Health type**	Focus area (eg, diseases)	N	Y	Y	N	Y
	Activity (eg, surveillance)	Y	Y	Y	Y	Y
**Source of information‡**						
**AidData**		N	Y (uses one of AidData’s Chinese Official Finance datasets)			N
**Government data: budget/financial accounts, yearbook statistics, information systems, websites, and/or reports**		Y	Y			Y
**Recipient country information system**		N	Y			N
**Academic literature**		N	Y			N
**Media reports**		Y	Y			N
**Interviews**		Y	N	N	N	N
**Data use**						
**Source verification process**		Notes that inconsistencies were double checked but process used unclear	AidData database adopts a ‘health of record score’ to signal which projects have the most complete and reliable data, in part based on a verification and triangulation process			No process identified
**Ranking system of sources**		No process identified	AidData database adopts a ranking system based on resource types, with official government sources at the top and media reports and social media at the lowest level			N/A only Chinese government data used
**Attempt to fill missing financial values**		No process identified	No process identified	Not attempted; focused on project counts instead of exact financial estimation	Substitutes median value of the same type of project for missing values	Uses a variety of estimation and regression models to fill in gaps

*This category attempts to align with key dimensions of the OECD Creditor Reporting System Aid Activity Database (CRS). Additionally, selected categories were also based on common categorizations used by datasets, like AidData’s Chinese Official Finance dataset. Three of the five studies in the table used an AidData dataset. AidData also attempts to align with OECD CRS standards, although to do so, some additional terminology unique to AidData, such as ‘vague’ finance or ‘pledges’, is included.

†Projects that are ‘in implementation’ or ‘completed’ are included in this analysis. Inclusion of these two project types could be considered a proxy for disbursements. This study uses one of AidData’s Chinese Official Finance datasets, which tracks a project’s status but does not explicitly track disbursements. In this dataset, a project is considered to be ‘in implementation’ if any portion of the intended amount has been disbursed. A project that is ‘completed’ assumes all committed finances have been disbursed.

‡Three of the five studies used a common data source (AidData’s Chinese Official Finance). To report consistently across categories, when a source was not specifically mentioned in an estimate, we defaulted to using AidData’s description of their source data. Some studies opted to include additional data to supplement their studies (eg, multilateral contributions data).

- *Definition and scope of health aid*: there are several differences in what is captured in the estimates: financial flows (ie, official development assistance and/or other official finance), the type of flows (ie, disbursements and/or commitments), the definition of health aid (ie, inclusion of allied sectors to health like water, sanitation and hygiene) and funding channels (ie, bilateral and/or multilateral).
- *Geographic scope*: only two of the five identified estimates are global. Three estimates focus primarily on Africa, which is most likely due to the timing of their publication. Before 2017, AidData, a common source for project-level Chinese aid data, had only released an Africa-specific database.
- *Time:* each dataset varies on reported years of data, with some starting as early as 2000 and others reporting figures as recently as 2017.
- *The level of disaggregation of results:* financial flows are reported in various ways: by channel (bilateral, multilateral, disbursing agency), by recipient country, and/or by project count. Each study adopts a different approach to categorising and understanding health focus areas (eg, diseases) and activity types (eg, surveillance), some of which attempt to align with OECD reporting requirements or with the Institute for Health Metrics and Evaluation’s aid classification system.
- *Data sources:* estimates vary on the degree to which available Chinese government data is supplemented with data from other sources, such as media reports, recipient budget documents or academic studies.
- *Approaches to using data:* estimates adopt varying approaches to handling missing data points, weighting some sources more than others when presented with conflicting evidence and triangulating conflicting information.

In the absence of official data, each estimate provides unique insights about the size, scope or focus of China’s health aid footprint. Together they provide a window into understanding China’s health portfolio. Despite differences in Chinese health aid estimations, these studies suggest that China is among the top 10 bilateral global health donors.

## What these estimates mean for the future

Although the findings of these studies may be helpful for showing directional trends, caution should be used when reporting or comparing these estimates given their diverse methodological approaches. Each study makes tradeoffs, and the use of a particular estimate will likely vary depending on the user’s requirements. Until China publicises its data, analysts and researchers need to understand the complexities of the Chinese aid ecosystem when choosing a methodological path or forming substantive conclusions. The details may seem highly technical but they are important for understanding a complex donor.

Among current estimates, and in the media more broadly, ‘aid’ and other types of ‘development finance’ are often conflated. Different instruments are used for different purposes, by China as well as by other donors. The lines between financing instruments are often blurred, and therefore estimates can overstate the boundaries of Chinese ‘aid’. Adhering to commonly used definitions, such as ‘official development assistance’, and norms, such as common standards for reporting aid for health or categorisation of health focus areas, could support a better snapshot of China’s portfolio.^7 8^ Researchers who track Chinese health aid could learn from the experience of researchers who track aid for women’s and children’s health, who recently collaborated on an exercise to agree on a common tracking methodology; the tracking of Chinese aid for health may benefit from a similar exercise.^9^ Such an exercise should, to the extent possible, ensure comparability with health aid from other donors.

The establishment of a new Chinese aid agency (China International Development Cooperation Agency, or CIDCA), presents many new opportunities. Although data transparency may not be a top priority for the new agency, such transparency could not only allow for improved tracking, but could help minimise some of the reputational risks it has incurred with development finance in general (eg, claims of predatory lending practices, also coined ‘debt-trap diplomacy’).^10^ Other donors could, and should, help support CIDCA’s capacity to make its aid data more transparent. However, until official data are made available, enhanced estimates for health aid could enable both fellow donors and recipient countries to allocate resources more efficiently. A better understanding of China’s health aid portfolio could also lead to improved collaboration among health donors.

## Conclusion

China has become a major player in the global development financing landscape. But due to limited data transparency, there are significant differences in the methodological approaches used to generate existing estimates of health aid from China. And so, it is important to exercise caution when comparing and reporting these estimates. Coherent approaches to estimating Chinese aid for health could improve alignment between China and other donors and allow for more efficient resource allocation by both fellow donors and recipient countries. Further, enhanced data transparency could also help mitigate some of the reputational risks that China has incurred recently. However, despite differences and room for growth, the existing estimates paint a consistent picture: China’s role in global health is no longer ‘emerging’. It has arrived.
